# Blue rubber bleb nevus syndrome: our experience and new endoscopic management

**DOI:** 10.1097/MD.0000000000007792

**Published:** 2017-08-18

**Authors:** Wenguo Chen, Hongtan Chen, Guodong Shan, Ming Yang, Fengling Hu, Qi Li, Lihua Chen, Guoqiang Xu

**Affiliations:** a Department of Gastroenterology, The First Affiliated Hospital, College of Medicine, Zhejiang University, Hangzhou, Zhejiang Province; b Department of Nephrology, Jilin City Central Hospital, Jilin, Jilin Province, China.

**Keywords:** blue rubber bleb nevus syndrome, endoscopic submucosal dissection, endoscopy, gastrointestinal, small bowel

## Abstract

The aim of our study is to enhance the awareness of blue rubber bleb nevus syndrome (BRBNS) through the patients in our hospital and introduced a new measure of endoscopic intervention.

A retrospective review of 5 patients, who were diagnosed as BRBNS in our hospital from January 2013 to January 2017, was conducted. Data were collected with regard to demographics, clinical presentation, endoscopic and imaging findings, management, and follow-up data.

In total of 5 patients, the mean age was 28.8 years, range 16 to 44 years (male/female, 1/4) with the average initial age of onset 15.4 years. No family history was identified in our group. Physical examination showed multiple cutaneous lesions in 2 patients (40%, 2/5). All the 5 patients had gastrointestinal tract vascular malformations; stomach involved in 2 cases, large intestine in 2 cases, and small intestine involved in 3 cases. Lesions in the visceral organs and tissue were found in 1 patient. Gastrointestinal bleeding was its main symptom (3/5, 60%). Laboratory investigations revealed anemia in 4 patients and abnormality of coagulopathy in 2 patients with severe anemia. Conservative approach was recommended in 3 cases that included iron supplementation, drug hemostasis, and/or blood transfusion. An innovatively therapeutic approach with endoscopic submucosal dissection (ESD) procedure was used successfully in 1 patient with 2 polypoid BRBNS lesions in rectum.

BRBNS is a very rare vascular malformation syndrome with unclear etiopathogenesis and noncurative treatments. ESD procedure was a feasible approach to remove the partial gastrointestinal lesions.

## 1 Introduction

Blue rubber bleb nevus syndrome (BRBNS), also known as the Bean syndrome, is a very rare disorder characterized by multiple vascular malformations of the skin, gastrointestinal (GI) tract, and, less often, other visceral organs.^[1]^ Typically, cutaneous lesions are usually asymptomatic, easily compressive and refill slowly upon release of pressure, and bleed spontaneously very rarely but easily upon being traumatized.^[2]^ To the clinicians, gastrointestinal bleeding and secondary iron deficiency anemia were the most common symptoms ascribed to the GI tract lesions. The onset of the disease could be mostly traced from birth, infancy, or early childhood; only few patients started after adulthood, and the lesions increased in size and number with the advancing age.^[3]^ However, due to its low incidence and atypical clinical symptoms, it was easily misdiagnosed in the early stage. To date, only few hundreds of cases have been reported since William Bennett Bean brought the BRBNS to our attention.^[4]^

Up to now, more and more literatures across the world have described this rare syndrome; nevertheless, the majority of the articles were case reports. In our manuscript, we retrospectively reviewed 5 patients admitted to our hospital who had been diagnosed with BRBNS in the 4 years between 2013 and 2017. We mainly analyzed their clinical characteristics, diagnosis, endoscopic and imaging findings, and treatment to enhance the awareness of the physicians. In addition, we reported a new therapeutic approach through endoscopic submucosal dissection (ESD), which was first to be reported to our knowledge.

## 2 Methods

From January 2013 to January 2017, a total of 5 BRBNS patients were found in our hospital at a mean age of 28.8 years, range 16 to 44 years (male/female, 1:4). The symptoms included the following: melena in 1 case, hematochezia in 1 case, abdominal pain and nausea in 1 case, fatigue in 1 case, and positive of fecal occult blood in 1 case. The initial ages of onset in the 5 patients were 7, 9, 14, 15, and 32 years, respectively, with the average age of 15.4 years. In the past, 2 patients had the surgical operation for removal of venous malformations: in one patient it was in colon, and in the other, it was located in lung. The long-term iron supplementation and occasional blood transfusion had been tried in 2 patients for many years before hospitalization. Multiple painless bluish nodular cutaneous lesions were found in 2 patients; physical examination revealed multihemangiomas appeared in the skin of trunk, limbs, tongue, etc. The other 3 patients had no skin lesions. There was no family history identified in our 5 cases. Table 1 listed the patients’ main characteristics.

**Table 1:**
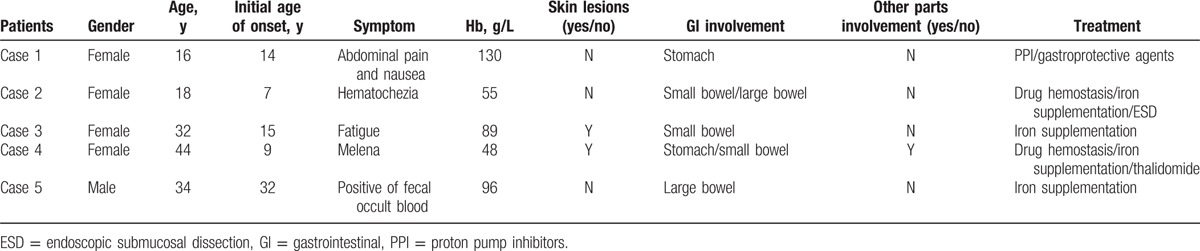
Patient characteristics.

We obtained human subjects approval from Ethics Committee of The First Affiliated Hospital, College of Medicine, Zhejiang University (Reference Number 2017-389). The record/information of patients were anonymized and de-identified prior to analysis. Our institutional review board approved this study with a waiver of informed consent.

## 3 Results

### 3.1 Endoscopic view

Both gastroscopy and colonoscopy were performed in all 5 patients: 3 patients finished the whole GI screening with capsule endoscopy (CE) examination. The locations of hemangiomas were listed as follows: 1 case only in stomach; 1 case in duodenum, jejunum, and rectum; and 1 case in jejunum and ileum. The other 2 patients, whose lesions were detected in stomach and in colon, respectively, were not examined by CE or double balloon enteroscopy (DBE), so their small bowel could not be evaluated endoscopically. However, abdomen computed tomography (CT) showed the small bowel was involved in 1 of these 2 patients.

**Figure 1:**
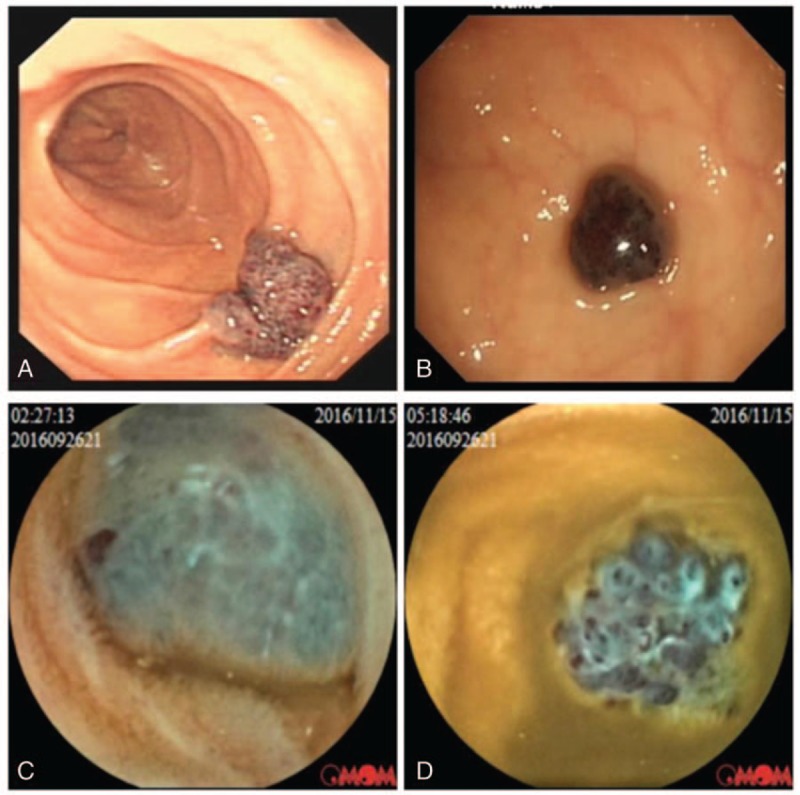
Gastroscopy (A), colonoscopy (B), and CE (C,D) examination showed multiple blue vascular nodular lesions in the gastrointestinal tract. (A, the descending part of the duodenum; B, rectum; C, jejunum; and D, ileum.) CE = capsule endoscopy.

**Figure 2:**
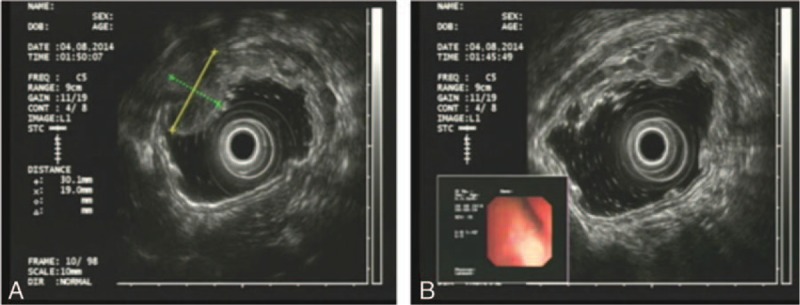
EUS revealed obvious thickening of the submucosa and muscularis propria with hypoechoic lesion (A), lesions with multiple hypoechoic and luminal structure (B) of various sizes in 1 patient of gastric vascular malformations. EUS = endoscopic ultrasonography.

Generally, endoscopic examination would show multiple, congested, bluish, tender, nodular hemangiomas in the GI tract. Clinically, various appearances may be present: sometimes they appear as strawberrylike mucosal polypoid, but have a submucosal protrusive lesion at the other times. The typical endoscopic images of BRBNS were shown in Figure 1. With the examination of endoscopic ultrasonography (EUS), the typical lesions were hypoechoic, with luminal structure predominantly involving the mucosa and submucosa. Muscularis propria, sometimes the whole layer, could be affected. In our paper, the EUS results of 1 patient with gastric multivascular malformations are presented in Figure 2.

### 3.2 Ancillary examination

Laboratory investigations revealed anemia in 4 patients, whose hemoglobin levels at the time of admission were 48, 55, 89, and 96 g/L, respectively. The red blood cells were microcytic and hypochromic, which indicated iron deficiency anemia. Laboratory tests also showed abnormality of coagulopathy, presented as the elongation of prothrombin time (PT), activated partial thromboplastin time (APTT), and the raised d-dimer in the 2 patients with severe anemia, whereas the concentration of fibrinogen decreased. The hemoglobin level was normal in 1 patient with abdominal pain and nausea, the fecal occult blood was also negative. No abnormality of coagulopathy was found in this patient.

**Figure 3:**
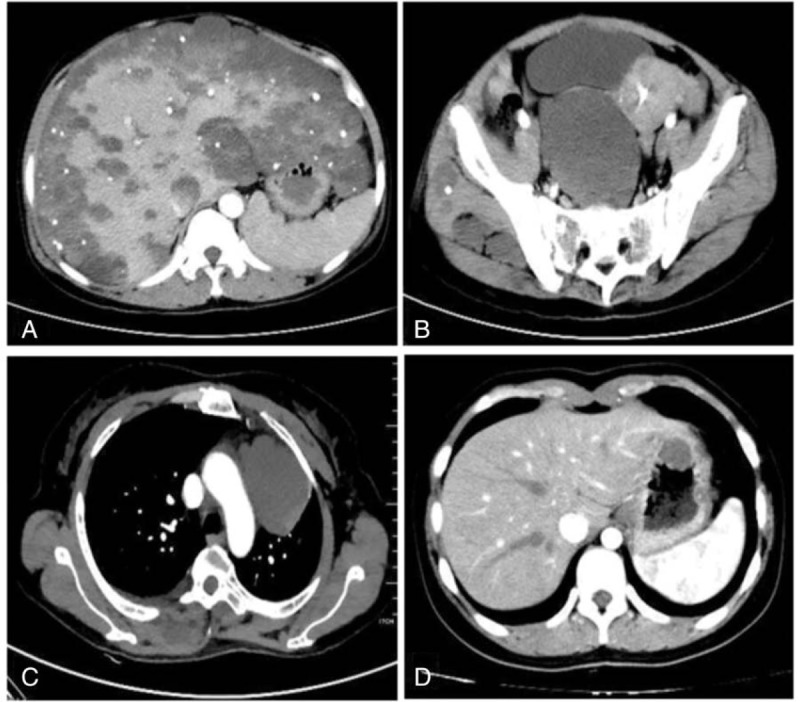
Abdomen CT scan with intravenous contrast administration revealed multiple vascular malformations on the liver (A), muscles of buttocks and pelvic cavity (B), mediastinum (C), and stomach (D), and multiple punctate calcification could be seen in the lesions. CT = computed tomography.

All of these 5 patients received the CT scan of the abdomen, which may be characterized by the thickening of bowel wall, nodular or irregular elevated lesions, soft-tissue masses in the GI, or abdominal cavity or viscera; and calcification of the hemangioma could be seen in partial cases. Enhancement in venous phase was sometimes observed through the inspection of the contrast-enhanced CT images. CT may help to identify extraintestinal lesions also. In our group, 1 patient revealed multiple vascular malformations on the liver, mediastinum, pelvic cavity, abdominal cavity, muscles of buttocks, and iliacus. However, all of the lesions were low density with no enhancement but multipunctate calcifications (Fig. 3). CT scan of head was advised to the 5 patients to exclude brain hemangiomas, which could cause fatal cerebral hemorrhage. Fortunately, we found no evidence of lesions in their CT results.

### 3.3 Treatment

**Figure 4:**
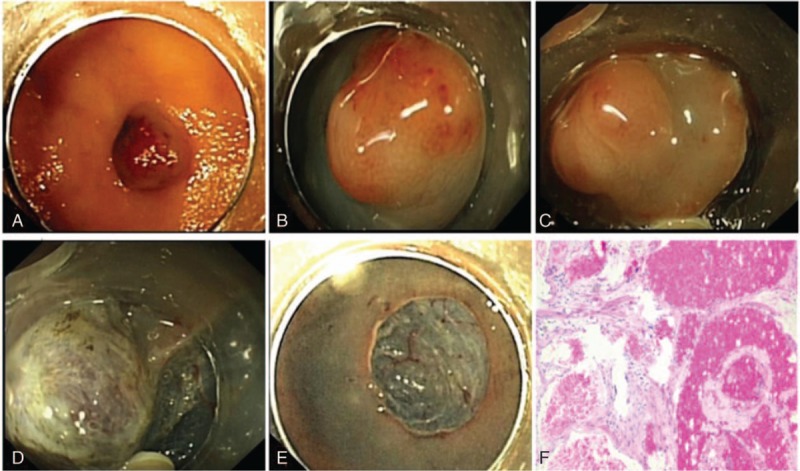
Endoscopic views of ESD procedure in a patient with rectum vascular malformation. (A) A 0.6 × 0.8 cm lesion located in the rectum. (B) Submucosal injection to lift the lesion. (C) Circumferential incision was performed. (D) Submucosal dissection. (E) ESD was finished and artificial ulcer was seen. (F) Histological examination showed dilated and hyperplasia capillaries in the lamina propria of mucosa. ESD = endoscopic submucosal dissection.

In our group, in 1 case admitted for repeated hematochezia, 2 polypoid BRBNS lesions, sized 0.6 × 0.8 and 0.5 × 0.6 cm, respectively, were detected in the rectum. To resect completely, we adopted innovative endoscopic measures—ESD in BRBNS, which was extensively used in the resection of early gastric cancer (EGC), gastrointestinal stromal tumors (GIST), etc. The ESD procedure was similar to other operations of GI tumors, which contained lesion marker, submucosal injection, circumferential incision, submucosal dissection, wound treatment, etc (Fig. 4). Lifting sign was helpful to judge whether the lesion involved the muscularis propria; EUS could also give these results. After the submucosal injection, the color of the lesion would turn lighter which may be ascribed to the compression and dilution of the injection fluid that was a feature of ESD in BRBNS. No bleeding happened in the procedure and the 2 lesions were resected successfully. Histologically, microscopic examination showed dilated and hyperplasia capillaries in the lamina propria of mucosa.

Conservative approach was recommended in the other 3 cases, which included iron supplementation, drug hemostasis, and/or blood transfusion. One patient with multigastric BRBNS, who chiefly complained of abdominal pain and nausea, showed negative fecal occult blood in laboratory tests, and had normal hemoglobin level, accepted the drug of proton pump inhibitors (PPI) and gastroprotective agents. All of the 5 patients were told to eat soft foods for rest of their lives. No antiangiogenic agents were used in our group because of the uncertain response. No massive sudden hemorrhage occurred, so surgery was not available.

### 3.4 Follow-up

After a minimum follow-up of 6 to 12 months in the 4 patients with anemia, 2 patients demonstrated a favorable response to iron supplementation; the hemoglobin level increased obviously (89–110 and 96–115 g/L, respectively). One patient with multigastric and small bowel lesions remained melena repeatedly after her discharge from the hospital; the hemoglobin level was still in lower level, laparotomy was refused by surgeon because of its extensive involvement in GI. So thalidomide treatment was administered to the patient after her informed consent. The case with ESD in the rectum had no GI bleeding again during the follow-up time within half a year; the hemoglobin level increased from 55 to 70 g/L. Another patient with multigastric BRBNS showed no melena or positive fecal occult blood.

## 4 Discussion

William Bennett Bean brought the BRBNS to our attention and named the condition in 1958, although Gascoyen had described it almost 100 years previously.^[5]^ The etiology and pathogenesis of this syndrome remained unknown; locus on chromosome 9P, elevated c-kit expression may be involved on molecular and genetic level.^[6]^ Although an autosomal dominant inheritance had been described in several familial cases, the majority of the cases appeared to be sporadic^[7]^; no family history of BRBNS was found in our group also. The venous malformations were primarily located on the skin and GI tract. Xue-Li Jin reviewed 120 cases from the literature; cutaneous angiomas were observed in 112 cases, which accounted for 93%, and GI lesions were observed in 76% cases.^[3]^ In our group, skin lesion appeared in 2 patient (40%), GI lesions were detected in all the 5 patients (100%). There seemed to be a positive correlation between the number of GI lesions and the number of cutaneous hemangiomas.^[8]^ In the reviewed cases, other parts of the body may have been affected, such as liver, spleen, pancreas, kidney, bladder, eye, brain, heart, lung, muscle, peritoneal cavity, thyroid, thymus, submandibular and parotid regions, adrenal gland, and joints.^[9,10]^

Skin lesions were usually observed through physical examination that could appear anywhere on the body but predominantly occurred on the trunk and upper limbs. The characteristic cutaneous lesions consisted of deep blue or purple, soft, rubbery blebs, easily compressible and turned to white; they may also lie deep in the skin and appear as bluish macules. Cutaneous lesions were presented in 2 patients in our study, multihemangiomas appeared in the skin of trunk, limbs, tongue, buttocks, etc. Complaint of painful lesion was reported in few patients because of the contraction of smooth muscle fibers surrounding the angioma.^[11]^ Skin lesions were seldom treated unless they were cosmetically unacceptable or functionally troublesome. Several studies have shown effective methods for the treatment of cutaneous lesions like laser, surgical removal, electrodesiccation, cryotherapy, and sclerotherapy.^[12]^ No treatment was intervened to the skin lesions in the 2 patients in our hospital because of the absent symptoms.

For BRBNS-involved GI tract, endoscopy was the first-line diagnostic tool and it enabled immediate therapeutic measures, especially in bleeding patients. Gastroscopy, colonoscopy, and CE were recommended for every BRBNS to screen the whole GI tract. Usually, the GI lesions presented at a later age but continued throughout life; the GI bleeding mainly manifested as intermittent melena, and sudden massive hemorrhage rarely occurred. Rarely, abdominal pain, intussusception, volvulus, infarction were its complications.^[13,14]^ On the morphological aspects, just like Kang Kook Choi's report, GI BRBNS could arise in diverse forms, including polylobulated, nodular, sessile, pedunculated, or ulcerated,^[15]^ and submucosal protrusive was also its appearance in our patients. Venous malformations could distribute throughout any segment of GI tract from the oral mucosa to the anal canal and mostly located in small intestine and distal colon. Some literatures also reported small intestine was the most frequently involved extracutaneous organ.^[15]^ In our 5 cases, gastric hemangioma was found in 2 cases (40%) and large intestine hemangioma in 2 cases (40%). In 3 cases (60%), small intestine was involved through CE and abdomen CT. With the examination of EUS, we could detect the typical hypoechoic lesions with luminal structure predominantly involving the mucosa and submucosa. Muscularis propria, even the whole layer could be affected. The transmural nature of lesion was reported in partial case reports based on the operation results, which might reveal the infeasibility and high-risk of the endoscopic intervention in partial BRBNS lesions.^[16]^

A diagnosis of BRBNS was made on the basis of the typical skin lesions or GI lesions. Ancillary examination was helpful in severity evaluation. The laboratory test may reveal anemia, abnormality of coagulopathy, and positive fecal occult blood. The anemia was characterized by hypochromic microcytic anemia with iron deficiency, all that indicated iron deficiency anemia. Abnormality of coagulopathy, which presented as the rise of PT, APTT, D-dimer, and decrease in the concentration of fibrinogen, was found in our patient with severe anemia. In addition, thrombocytopenia, or chronic lymphocytic leukemia may occur in literature.^[17]^ CT examination especially contrast-enhanced CT was the most valuable noninvasive screening procedure in BRBNS which could identify GI hemangiomas and extra intestinal lesions. On the contrast-enhanced CT images, the lesions were demonstrated by the thickening of bowel wall, nodular or irregular elevated lesions, soft-tissue masses, and enhancement in venous phase. Systemic complications, such as thrombosis and the calcification of vascular lesions could also be observed through CT. In one of our patient, low-density lesions with no obvious enhancement but multipunctate calcifications were located in liver, mediastinum, pelvic cavity, abdominal cavity, muscles of buttocks, and iliacus. Like in Vig EK's report,^[18]^ it was important to examine the sites of possible vascular malformations as a routine measure, especially in the central nervous system. We suggested CT or magnetic resonance imaging (MRI) of brain was necessary to exclude brain hemangiomas that involved fatal cerebral hemorrhage. CT could also be helpful in evaluating the appeared abdominal complications and made some preliminary location judgments in active GI bleeding of BRBNS.

Nowadays, although a wide range of therapeutic options had been described in the literature, there was no consensus on the treatment of the GI tract vascular malformations. The prognosis and treatment depended on the extent of GI tract involvement and the symptoms at presentation. Diet was important to the GI BRBNS patients; soft food was therefore required particularly for patients with the history of GI bleeding. In accordance with reports,^[19]^ drug hemostasis and iron supplementation had good clinical outcomes in our partial patients with mild bleeding. Antiangiogenic agents, such as corticosteroids, propranolol, thalidomide, and interferon-a, and immunosuppressive agents like sirolimus had been described in treating vascular lesions and lessening the frequency of bleeding.^[20]^ The majority of therapies were only limited to case reports which lacked long-term prospective data. In our study, 1 case adopted the pharmacological therapeutic strategies of thalidomide because of the recurrent gastrointestinal bleeding in the period of follow-up. Progress in endoscopic technology had advanced medical practice, endoscopic interventions such as argon plasma coagulation, band ligation, endoloops, or lauromacrogol injection (sclerotherapy) had been suggested for the treatment of vascular lesions, and good clinical outcomes had been reported.^[21]^ Lesions located in small intestine and transmural lesions were relatively formidable and had high risk of perforation that should be viewed with great caution. To date, the role of surgical resection in the management of GI BRBNS was deemed controversial. In Hasan Yuksekkaya's literature, surgical resection had been condemned as overly aggressive and unhelpful because of the belief that lesions would recur after removal^[22]^; the risk of short bowel syndrome should be considered if the GI lesions were extensive. Conversely, Zhao-Hui Deng's^[23]^ review described surgical wedge excision of angiomas or segmental resection of involved bowels was recommended in case of severe or recurrent hemorrhage, and surgery combined with intraoperative endoscopy intervention was proposed. We also thought surgical resection was essential in patients with recurrent bleeding, and complete visualization of the entire GI was necessary before operation through gastrointestinal endoscope. Staged operative procedures may require in patients with an overwhelming number of lesions.^[24]^ In the postoperative period, antiangiogenic agents or immunosuppressive agents could also be tried to prevent the recurrence of bleeding.

As we all know, ESD had launched a new era of broadened indication for endoscopic resection especially in early GI cancer, GIST, leiomyoma, laterally spreading tumor (LST), etc. Here we reported a new therapeutic approach using ESD in BRBNS lesions, which had not been reported because of its low incidence and high risk. Two rectum vascular malformations were resected completely in our center without intraoperative and postoperative complications. The ESD procedure was carried out in a standardized traditional way. EUS combined with intraoperative lifting-sign was helpful in pointing out the involved layer of the lesions to avoid transmural BRBNS. Because of its hemangioma characteristics, like the skin lesions, the lesion's color would turn lighter after the submucosal injection of the mixture with glycerol fructose solution, indigo carmine, and 0.001% epinephrine. In accordance with literature,^[25]^ histologically microscopic examination showed dilated and hyperplasia capillaries in the lamina propria of mucosa consistent with cavernous hemangiomas. The complete resection of lesions may reduce the recurrence of new angiomas compared with other endoscopic managements and increased the quality of life. However, ESD for BRBNS also had its limitations like small bowel lesion, transmural lesion, lesions with widely involvement. So we suggested the gastric and colonic BRBNS located above the submucosa, polypoid lesion, and single or less than 5 lesions were the main indications. Lesions located in the duodenal bulb and the descending part of the duodenum could also be tried.

In summary, BRBNS is a very rare vascular malformation with unclear etiopathogenesis and noncurative treatments. The diagnosis relied to the typical skin lesions and/or GI lesions. A wide range of therapeutic options including medical management, endoscopic intervention, and surgical resection had been proposed. However, sufficient evidence, long-term prospective data, and new available therapies remain to be searched in the future. Meanwhile, we deduced that ESD procedure is a feasible approach to remove the partial GI lesions. Because of the recurrences and incurable characteristics of GI hemangioma, every patient should be counseled about the chronicity of this disease and regular follow-up throughout their life.
